# Supplementary material for: Chemophenetic Approach to Selected Senecioneae Species, Combining Morphometric and UHPLC-HRMS Analyses
Source: Plants (Basel). 2023 Jan 14;12(2):390. doi: 10.3390/plants12020390 (PMC9866700; doi:10.3390/plants12020390)
Supplement: Supplementary file 1 [file plants-12-00390-s001.zip › Tables S2-S4, Figure S1.pdf]

Supplementary Tables (S2-S4) and Figure S1

Table S2. Descriptive statistics on the morphometric data. SD – standard deviation; CV% coefficient of variation (relative SD) in percentages; CI – confidence interval at 95%.

	J.maritima (N=15)	*J.pancicii* (N=15)	*S.hercynicus* (N=15)	*S.ovatus* (N=15)	*S.rupestris* (N=15)
x1					
- Mean (SD, CV%)	0.36 (0.05, 14%)	0.37 (0.07, 19%)	0.43 (0.05, 12%)	0.47 (0.05, 11%)	0.47 (0.05, 11%)
- Range	0.30–0.40	0.30–0.50	0.40–0.50	0.40–0.50	0.40–0.50
- Mean (CI)	0.36 (0.33, 0.39)	0.37 (0.33, 0.41)	0.43 (0.41, 0.46)	0.47 (0.45, 0.50)	0.47 (0.44, 0.49)
x2					
- Mean (SD, CV%)	55.4 (10.33, 19%)	41.93 (4.22, 10%)	79.27 (4.68, 6%)	70.83 (13.97, 20%)	54 (3.91, 7%)
- Range	35.00–70.00	30.00–48.00	72.00–89.00	35.00–87.00	47.00–59.00
- Mean (CI)	55.40 (49.68, 61.12)	41.93 (39.60, 44.27)	79.27 (76.67, 81.86)	70.83 (63.10, 78.57)	54.00 (51.83, 56.17)
x3					
- Mean (SD, CV%)	8.95 (1.38, 15%)	10.11 (2.16, 21%)	13.71 (1.16, 8%)	10.94 (1.46, 13%)	7.31 (1.16, 16%)
- Range	6.80–11.00	7.00–14.50	12.00–17.00	9.00–14.00	5.50–9.60
- Mean (CI)	8.95 (8.18, 9.71)	10.11 (8.92, 11.31)	13.71 (13.06, 14.35)	10.94 (10.13, 11.75)	7.31 (6.66, 7.95)
x4					

- Mean (SD, CV%)	4.44 (0.78, 18%)	2.65 (0.63, 24%)	3.33 (0.29, 9%)	3.19 (0.5, 16%)	2.87 (0.74, 26%)
- Range	3.00–6.00	1.90–4.00	2.90–3.80	2.40–4.00	2.00–4.00
- Mean (CI)	4.44 (4.01, 4.87)	2.65 (2.30, 3.00)	3.33 (3.17, 3.49)	3.19 (2.91, 3.46)	2.87 (2.46, 3.28)
x5					
- Mean (SD, CV%)	0.61 (0.03, 5%)	0.74 (0.08, 11%)	0.74 (0.05, 7%)	0.76 (0.11, 14%)	0.55 (0.05, 9%)
- Range	0.55–0.65	0.60–0.90	0.70–0.80	0.60–0.90	0.50–0.60
- Mean (CI)	0.61 (0.59, 0.63)	0.74 (0.69, 0.79)	0.74 (0.71, 0.77)	0.76 (0.70, 0.82)	0.55 (0.52, 0.58)
x6					
- Mean (SD, CV%)	12.73 (0.96, 8%)	18.2 (1.26, 7%)	10.53 (1.77, 17%)	10.6 (1.24, 12%)	13.4 (1.99, 15%)
- Range	12.00–15.00	16.00–21.00	8.00–13.00	9.00–14.00	11.00–16.00
- Mean (CI)	12.73 (12.20, 13.27)	18.20 (17.50, 18.90)	10.53 (9.55, 11.51)	10.60 (9.91, 11.29)	13.40 (12.30, 14.50)
x7					
- Mean (SD, CV%)	0.46 (0.07, 15%)	0.83 (0.12, 14%)	0.73 (0.07, 10%)	0.97 (0.12, 12%)	0.55 (0.05, 9%)
- Range	0.30–0.55	0.70–1.10	0.60–0.80	0.80–1.20	0.50–0.60
- Mean (CI)	0.46 (0.42, 0.50)	0.83 (0.76, 0.90)	0.73 (0.69, 0.77)	0.97 (0.91, 1.04)	0.55 (0.52, 0.58)
x8					
- Mean (SD, CV%)	12.73 (1.28, 10%)	16.93 (2.25, 13%)	6.53 (0.52, 8%)	5.07 (0.26, 5%)	11.33 (0.49, 4%)
- Range	11.00–15.00	12.00–20.00	6.00–7.00	5.00–6.00	11.00–12.00

- Mean (CI)	12.73 (12.02, 13.44)	16.93 (15.69, 18.18)	6.53 (6.25, 6.82)	5.07 (4.92, 5.21)	11.33 (11.06, 11.60)
x9					
- Mean (SD, CV%)	0.72 (0.06, 8%)	0.8 (0.11, 14%)	0.85 (0.06, 7%)	0.71 (0.08, 11%)	0.53 (0.05, 9%)
- Range	0.60–0.80	0.60–1.00	0.80–1.00	0.60–0.90	0.50–0.60
- Mean (CI)	0.72 (0.69, 0.75)	0.80 (0.74, 0.86)	0.85 (0.81, 0.88)	0.71 (0.66, 0.75)	0.53 (0.51, 0.56)
x10					
- Mean (SD, CV%)	40.6 (3.66, 9%)	39.27 (8.14, 21%)	11 (1.36, 12%)	11.73 (2.69, 23%)	33.73 (3.84, 11%)
- Range	35.00–47.00	23.00–56.00	9.00–14.00	8.00–19.00	28.00–40.00
- Mean (CI)	40.60 (38.57, 42.63)	39.27 (34.76, 43.77)	11.00 (10.25, 11.75)	11.73 (10.25, 13.22)	33.73 (31.60, 35.86)
x11					
- Mean (SD, CV%)	1.03 (0.12, 12%)	2.58 (0.28, 11%)	1.73 (0.1, 6%)	2.1 (0.26, 12%)	1.65 (0.09, 5%)
- Range	0.70–1.20	2.20–3.00	1.60–2.00	1.40–2.40	1.50–1.80
- Mean (CI)	1.03 (0.96, 1.10)	2.58 (2.43, 2.73)	1.73 (1.68, 1.79)	2.10 (1.96, 2.24)	1.65 (1.60, 1.70)
x12					
- Mean (SD, CV%)	52.67 (18.33, 35%)	6.2 (2.54, 41%)	48.27 (9.73, 20%)	44.2 (15.87, 36%)	23.33 (8.36, 36%)
- Range	28.00–79.00	3.00–13.00	35.00–70.00	19.00–79.00	8.00–36.00
- Mean (CI)	52.67 (42.52, 62.81)	6.20 (4.79, 7.61)	48.27 (42.88, 53.65)	44.20 (35.41, 52.99)	23.33 (18.70, 27.97)

X_1 - root diameter [cm], X_2 - stem height [cm], X_3 - leaf length [cm], X_4 - leaf width [cm], X_5 - involucral bract length [cm], X_6 - involucral bracts number per capitula, X_7 - ray flower length [cm], X_8 - number of ray flowers per capitulum, X_9 - disc flower length [cm], X_{10} - number of disc flowers per capitulum, X_{11} - flower head diameter [cm], X_{12} - number of capitula per plant.

Table S3. Selection of a model with n variables.

	X_1	X_2	X_3	X_4	X_5	X_6	X_7	X_8	X_9	X_{10}	X_{11}	X_{12}
1	*											
2	*								*			
3								*	*		*	
4	*							*	*		*	
5	*						*	*	*		*	
**6**	*****			*****			*****	*****	*****		*****	
7	*		*	*			*	*	*		*	
8	*	*	*	*			*	*	*		*	
9	*	*	*	*	*		*	*	*		*	
10	*	*	*	*	*	*	*	*	*		*	
11	*	*	*	*	*	*	*	*	*		*	*
12	*	*	*	*	*	*	*	*	*	*	*	*

An asterisk indicates that a given variable is included in the corresponding model. For instance, it was observed that the best one-variable model contains just X_1, the best two-variable model contains X_1+X_4, then X_1+X_4+X_9, etc. As mentioned before, a 6 variables model was selected.

Table S4. Prediction of membership of the test set (n = 15)

	J. maritima	*J. pancicii*	*S. hercynicus*	*S. ovatus*	*S. rupestris*
J. maritima	3	0	0	0	0
J. pancicii	0	2	0	0	0
S. hercynicus	0	0	4	1	0

S. ovatus	0	0	0	3	0
S. rupestris	0	0	0	0	2

The model achieves complete discrimination of all species, except the *S. ovatus* and *S. hercynicus* classes (observation 16 is falsely classified as belonging to the *S. hercynicus* class).

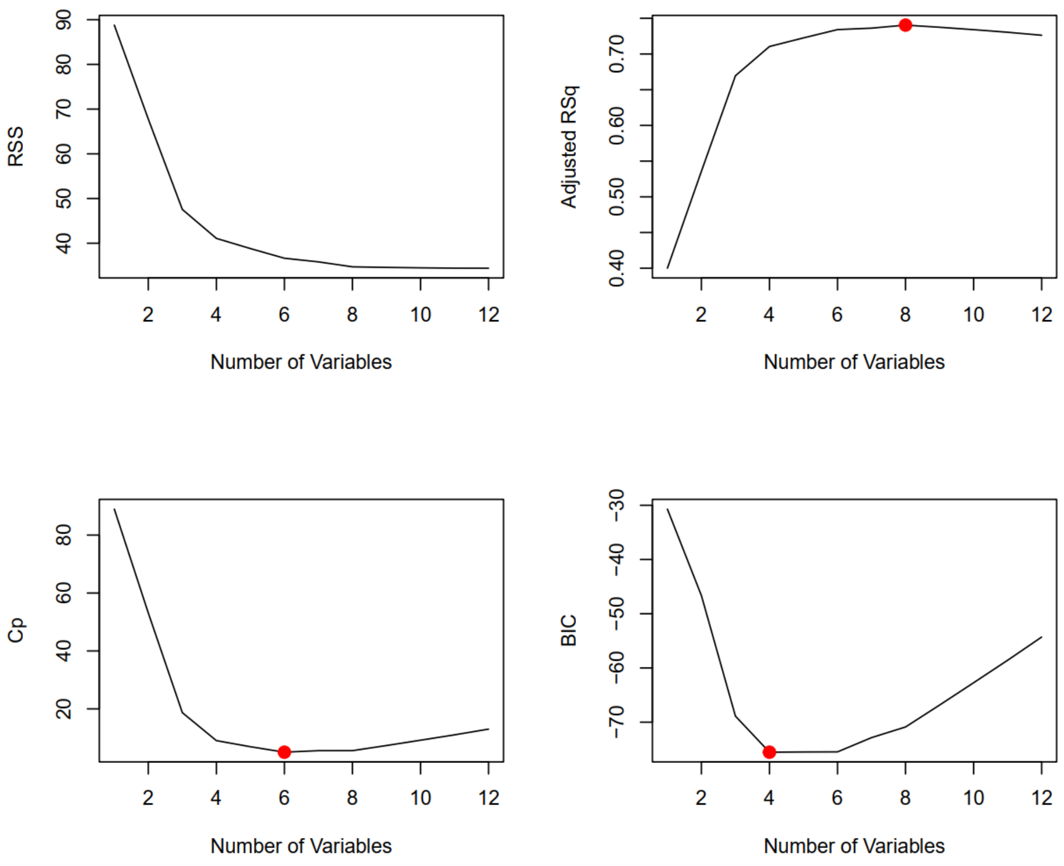

Figure S1. Residual sum of squares (RSS), adjusted R2, Mallow's Cp, and Bayesian information criterion (BIC) for the standardized morphological data.
